# The Methodological Quality of Studies Investigating the Acute Effects of Exercise During Hypoxia Over the Past 40 years: A Systematic Review

**DOI:** 10.3389/fphys.2022.919359

**Published:** 2022-06-16

**Authors:** Erich Hohenauer, Livia Freitag, Miriam Herten, Julia Siallagan, Elke Pollock, Wolfgang Taube, Ron Clijsen

**Affiliations:** ^1^ Rehabilitation and Exercise Science Laboratory (RES Lab), Department of Business Economics, Health and Social Care, University of Applied Sciences and Arts of Southern Switzerland, Landquart, Switzerland; ^2^ International University of Applied Sciences THIM, Landquart, Switzerland; ^3^ Department of Neurosciences and Movement Science, University of Fribourg, Fribourg, Switzerland; ^4^ Department of Movement and Sport Sciences, Vrije Universiteit Brussel, Brussels, Belgium; ^5^ Department of Physiotherapy, Zurich University of Applied Sciences, Zurich, Switzerland; ^6^ Department of Health, Bern University of Applied Sciences, Berne, Switzerland

**Keywords:** hypoxia, exercise, review, methodological quality, PEDro

## Abstract

Exercise under hypoxia and the physiological impact compared to normoxia or hypoxia has gained attention in the last decades. However, methodological quality assessment of articles in this area is lacking in the literature. Therefore, this article aimed to evaluate the methodologic quality of trials studying exercise under hypoxia. An electronic search was conducted until December 2021. The search was conducted in PubMed, CENTRAL, and PEDro using the PICO model. (P) Participants had to be healthy, (I) exercise under normobaric or hypobaric hypoxia had to be (C) compared to exercise in normoxia or hypoxia on (O) any physiological outcome. The 11-item PEDro scale was used to assess the methodological quality (internal validity) of the studies. A linear regression model was used to evaluate the evolution of trials in this area, using the total PEDro score of the rated trials. A total of *n* = 81 studies met the inclusion criteria and were processed in this study. With a mean score of 5.1 ± 0.9 between the years 1982 and 2021, the mean methodological quality can be described as “fair.” Only one study reached the highest score of 8/10, and n = 2 studies reached the lowest observed value of 3/10. The linear regression showed an increase of the PEDro score of 0.1 points per decade. A positive and small tendency toward increased methodologic quality was observed. The current results demonstrate that a positive and small tendency can be seen for the increase in the methodological quality in the field of exercise science under hypoxia. A “good” methodological quality, reaching a PEDro score of 6 points can be expected in the year 2063, using a linear regression model analysis. To accelerate this process, future research should ensure that methodological quality criteria are already included during the planning phase of a study.

## Introduction

Increased interest in altitude training as well as a popular trend toward reaching higher altitudes for sporting activities or traveling justifies the importance of understanding physiologic changes at higher altitudes, particularly during exercise. Several altitude classifications exist (Mazzeo, 2008; Dietz and Hackett, 2019). The most common of these is by Bärtsch and Saltin (2008): sea level is considered to lie between 0 and 500 m, low altitudes range from 500 to 2000 m, moderate altitude from 2000 to 3,000 m, high altitude above 3,000 m, and extreme above 5,000 m (Bärtsch and Saltin, 2008). Most mountain and ski resorts are located at a moderate altitude (Dietz and Hackett, 2019). Rapid ascent, representing acute hypoxic conditions, can lead to symptoms of altitude illness, which is the result of impaired acclimatization (Dietz and Hackett, 2019).

The main factor, associated with (acute) exposure to altitude is hypoxia, which is defined as tissue oxygen supply below the needed levels, to maintain normal physiological function (Loiacono and Shapiro, 2010). There are four main types of hypoxia, which can be classified into hypoxic hypoxia, anemic hypoxia, circulatory (stagnant or ischemic) hypoxia, and histotoxic hypoxia (Pittman, 2016; Cheung and Ainslie, 2022). The most common form of hypoxia is hypoxic hypoxia, which is the result of reduced arterial oxygen tension (Cheung and Ainslie, 2022). The physiologic response of the human body to hypoxia depends on the duration of exposure (short and long term), the magnitude of reduced ambient pressures, reduction in ambient oxygen pressure, the rate of occurrence, and severity of the exposure (Dietz and Hackett, 2019; Cheung and Ainslie, 2022). These physiological responses have been highlighted and explained in the literature (Michiels, 2004; Millet et al., 2012; Mounier and Brugniaux, 2012).

Hypoxic hypoxia can be the result of reduced barometric pressures at altitude, leading to a reduced partial pressure of inspired oxygen (Cheung and Ainslie, 2022). Barometric pressure decreases with increased terrestrial altitude, resulting in proportionally lower atmospheric oxygen partial pressures, while the oxygen percentage stays constant (20.9%) (Brown and Grocott, 2013). The direct consequences of lowered atmospheric oxygen partial pressure are a decrease in the partial pressure of oxygen in the body and blood tissues, a decrease in the arterial O_2_ partial pressure, and a reduction of the oxygen tension in the alveoli (Rodway et al., 2003; Sharp and Bernaudin, 2004). In the setting of hypobaric hypoxia and normobaric hypoxia, the respiratory ventilation is increased to compensate the reduced partial pressure of inspired oxygen.

The effects of altitude on human physiology were described as early as 1644. Evangelista Torricelli (1,608–1,647), a student of the great Galileo, was the first person to clearly state that the atmosphere exerts pressure. Over the proceeding years, many experiments with hypobaric and hyperbaric chambers and those at effective altitudes (e.g., balloon and mountain) were performed (West, 2016). Over the last few centuries, a large amount of scientific knowledge about altitude exposure was gained. Rapid exposure to high altitudes can result in acute hypoxia that affects many physiological systems, including the respiratory, cardiovascular, and neurologic systems (Cheung and Ainslie, 2022; Hohenauer, 2022). One of the most important responses of the body to hypoxia is to increase ventilation. As a result of lower partial pressure of oxygen, the increase in minute ventilation is triggered by oxygen-sensing cells in the carotid body (Dietz and Hackett, 2019). Further physiologic adaptions to altitude include an increased resting and sub-maximal heart rate, increased blood pressure, and decreased maximal oxygen consumption (Wyatt, 2014).

A considerable number of review studies about physical activity during hypoxia exposure have been performed over the past few decades (Ando et al., 2020; Pojskić et al., 2021), investigating its physiologic consequences (Coppel et al., 2015; Fernandez-Lazaro et al., 2019; Griffiths et al., 2019). Exercise training under hypoxia, as part of elite sports training, was established in the late 1960s, as it is advantageous compared to sea-level training to increase oxygen delivery capacity and aerobic exercise capacity (Park et al., 2016). Exercise under hypoxia is currently not only integrated into elite sports but also used in the field of health and rehabilitation (Nishiwaki et al., 2011; Kong et al., 2014; Schreuder et al., 2014). However, evidence from the literature shows that several methods and variables have to be taken into account during hypoxic training which determine its effectiveness (Millet et al., 2010). In general, exercise under hypoxia is associated with a compensatory increase in blood flow toward active muscles, resulting in pronounced shear stress and nitric oxide release (González-Alonso et al., 2006; Casey and Joyner, 2012). Exercise under hypoxia, therefore, seems to stimulate arterial remodeling/function and angiogenesis (Geiser et al., 2001; Ridnour et al., 2005; Tinken et al., 2010; Hellsten and Hoier, 2014). However, this is controversially discussed, with reports indicating superior (Geiser et al., 2001; Nishiwaki et al., 2011; Kon et al., 2015) or similar (Desplanches et al., 2014; Kong et al., 2014; Schreuder et al., 2014) vascular adaptations following hypoxic versus normoxic exercise. Debates on the difference between normobaric and hypobaric hypoxia highlight the increased interest and developments in this area (Millet and Debevec, 2020; Richalet, 2020).

Internal validity and external validity are the most relevant components when critically appraising randomized controlled trials although there is no gold standard method available (Jung et al., 2022). The internal validity of a study reflects the systematic error or bias in a clinical trial (Higgins and Green, 2011; Boutron et al., 2019), expressing the methodological robustness of a study (Jung et al., 2022). External validity is known by several definitions, and the terms generalizability, external validity, applicability, or transferability are used interchangeably in the literature (Weise et al., 2020). In an internally valid trial, external validity refers to the ability of the results to be generalized to the “real world” population (Akobeng, 2008). Consequently, a lack of internal validity adversely influences the quality of the evidence that can be derived from a trial. Without internal validity, an experiment cannot demonstrate a causal link between two variables. The main errors that could negatively affect the internal validity are bias (systematic error) and random error (chance error or statistical error) (Keirse and Hanssens, 2000; Stephenson and Babiker, 2000; Akobeng, 2008). Therefore, it is an important step to assess the methodological quality of trials, build an evidence base that informs clinical practice, and identify areas of healthcare that require further research (de Morton, 2009). In general, there are three types of tools for establishing internal validity: scales, checklists, and items (Jüni et al., 2001; Zeng et al., 2015).

Although there are many scales available that assess the methodological quality of clinical trials (Ma et al., 2020), the PEDro scale is commonly employed to assess the internal validity (Maher et al., 2003) and was already used in the field of hypoxia (Camacho-Cardenosa et al., 2019). The PEDro scale considers two aspects of trial quality, namely, the “believability” (or “internal validity”) of the trial and whether the trial contains sufficient statistical information to make it interpretable. It does not rate the “meaningfulness” (or “generalizability” or “external validity”) of the trial or assess the size of its treatment effect.

To the authors’ knowledge, no systematic review has evaluated the methodological quality of studies that investigated the effects of exercises in the setting of acute hypoxia on physiological parameters over the past 40 years. The present systematic review uses the PEDro scale to evaluate the methodological quality of studies that examined the effects of exercise under acute hypoxic conditions (normobaric or hypobaric) vs. normoxic conditions or acute hypoxic conditions under different barometric pressure on physiologic parameters and to assess the evolution of the methodological quality of these trials over the last 40 years.

## Materials and Methods

### Literature Search Strategies and Data Sources

A literature search was conducted using the PICO model from the PRISMA guidelines (Page et al., 2021): 1) Population: healthy, female and male study participants could be of any training status; 2) Intervention: exercise under hypobaric or normobaric hypoxia; 3) Comparator: exercise under normoxia or hypoxia; and 4) Outcomes: physiological parameters including, but not limited to heart rate, oxygen saturation (of the blood or muscle), blood flow, core temperature, blood markers, and respiration characteristics.

A systematic search was performed electronically until December 2021 in the following databases: MEDLINE (PubMed), Cochrane Central Register of Controlled Trials (CENTRAL), and Physiotherapy Evidence Database (PEDro), according to the PRISMA statement. The keywords and their combinations that were used in this work are shown in Table 1 *.*

**TABLE 1:** Screened databases, keywords, and identified studies.

Database	Keywords	Total Studies
PEDro	Hypoxia	72
PubMed	((((“hypoxia” [MeSH Terms] OR “hypoxia” [All Fields]) OR “hypoxia s” [All Fields]) OR “hypoxias” [All Fields]) AND (“hypobaric” [All Fields] OR “hypobarism” [All Fields])) OR “normobaric” [All Fields])	5,492
Cochrane trials	hypoxia AND normobaric OR hypobaric	879

### Selection Criteria

Eligibility criteria were based on the PICO approach. The following selection criteria were used: 1) all participants were healthy humans, 2) healthy participants had to perform any exercise under acute (<24 h) normobaric or hypobaric hypoxic conditions, 3) physiologic values were measured during and/or after exercise, and 4) only experimental studies were included. Studies were excluded in cases of 1) participants were exposed to hypoxia for longer than 24 h, 2) supplement (caffeine, vitamins, saline, etc.) or medication intake (e.g., formoterol, beta-blocker, sildenafil), 3) studies were published in a language other than English, or 4) no physical exercises were performed.

### Assessment of Methodological Quality Using the PEDro Scale

The German version of the PEDro scale was used to assess the methodological quality of the included trials. The German version of the PEDro scale demonstrated good inter-reliability for individual items and the total PEDro score (Costa et al., 2015). The PEDro scale, which is based on the Delphi list, is a valid (convergent and construct validity) and reliable tool for assessing trial methodologic quality (Maher et al., 2003; Macedo et al., 2010). The use of the PEDro scale, outside the classical field of physiotherapy, is growing (Elkins et al., 2013). This shows that its use is not limited to currently practiced methods in physiotherapy, nor that the trials have to be conducted by a physiotherapist (PEDro, 2017). The PEDro scale was used to assess the internal validity and has already been used in studies dealing with healthy participants (Fradkin et al., 2006; Ganio et al., 2009), patients (Pinto et al., 2012; Kouloutbani et al., 2019) and also in the field of hypoxia (Camacho-Cardenosa et al., 2019). The scale includes 11 items, but item 1 (eligibility criteria were specified) is not included in the calculation of the total score. A maximum of 10 points is therefore possible. A brief description of each PEDro item (English version) can be seen in Table 2. A PEDro score of 9–10 is considered to reflect an “excellent,” 6 to 8 a “good,” 4 to 5 a “fair,” and <4 a “poor” methodological quality (Cashin and McAuley, 2020).

**TABLE 2:** PEDro score.

Nr	Item	No	Yes	Where
1	Eligibility criteria were specified			
2	Subjects were randomly allocated to groups (in a crossover study, subjects were randomly allocated an order in which treatments were received)			
3	Allocation was concealed			
4	The groups were similar at baseline regarding the most important prognostic indicators			
5	There was blinding of all subjects			
6	There was blinding of all therapists who administered the therapy			
7	There was blinding of all assessors who measured at least one key outcome			
8	Measures of at least one key outcome were obtained from more than 85% of the subjects initially allocated to groups			
9	All subjects for whom outcome measures were available received the treatment or control condition as allocated or, where this was not the case, and data for at least one key outcome was analyzed by “intention to treat”			
10	The results of between-group statistical comparisons are reported for at least one key outcome			
11	The study provides both point measures and measures of variability for at least one key outcome			

A team internal briefing took place, during which each item of the PEDro score was discussed for reliability reasons. It was set forth, that a point for an item could only be awarded if the criterion was fulfilled. For Item 1, a list of inclusion criteria and exclusion criteria were required to fulfill the criterion. Furthermore, it had to be clearly described how the subjects were recruited. For Item 4, the trial must have performed a baseline measure of the severity of the condition being treated and at least one key outcome. In addition, it had to be shown that these parameters did not differ significantly between the different groups (e.g., through a *p*-value). Finally, for Item 11, both point measures and measures of variability were required.

### Data Extraction

**FIGURE 1:**
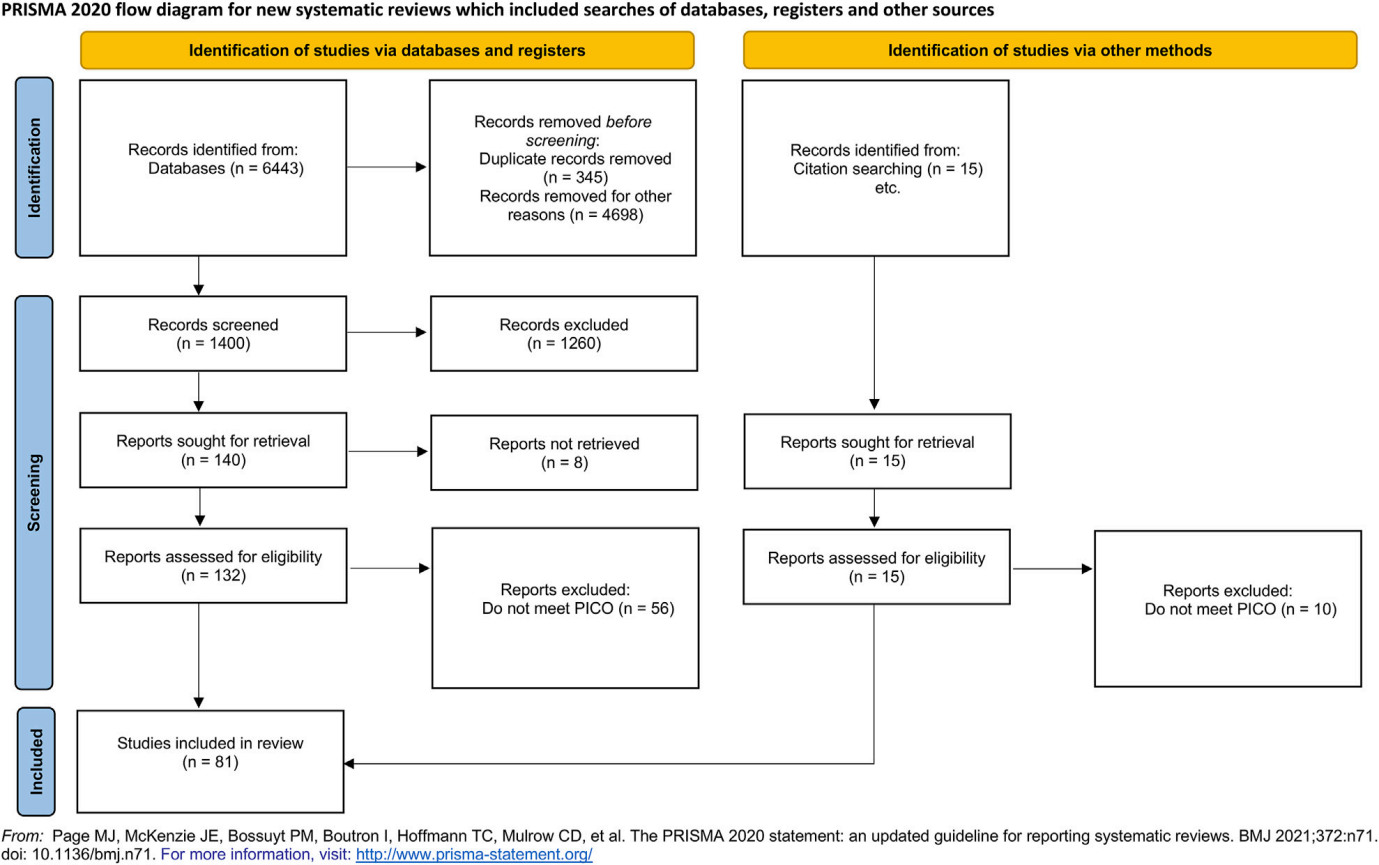
Flowchart describing the systematic selection procedure.

A total of *n* = 6,443 studies were identified from the main search strategy (Table 1) in databases and registers. After removing duplicates (*n* = 345) and for other reasons (*n* = 4,698), a total of *n* = 1,400 studies were used for the screening process. During this process, a total of *n* = 1,316 were excluded because they did not meet the PICO scheme or due to other reasons, and *n* = 8 reports were not retrieved. This resulted in the inclusion of *n* = 76 studies from databases and registers. A total of *n* = 15 studies were retrieved from citation searching. From these *n* = 15 studies, a total of *n* = 10 studies were extracted because they did not meet the defined PICO scheme, resulting in an inclusion of *n* = 5 studies from other methods. Figure 1 depicts the systematic search strategy and selection process.

Included articles were downloaded and saved in alphabetical order in a pdf format. The following variables were extracted: author names, article title, and publication year. Four researchers (EP, JS, LF, and MH) independently scored all trials (*n* = 81) for methodological study quality with the PEDro score (each item and the total score of the PEDro scale). Two researchers each rated the same article. In case of disagreement between the researchers, a third researcher rated the questionable item, and agreement was sought by consensus.

### Data Analysis

All data were analyzed using the Statistical Package for the Social Sciences (SPSS version 27.0, IBM, Armonk, United States). A bubble plot was created (DataGraph 4.7.2beta, Visual Data Tools Inc. Chapel Hill, United States) based on the total score of the PEDro scale (dependent variable) and the publication year (independent variable) of each study. The size of the bubble was dependent on the number of studies with the same total PEDro score for each year, to assess the relationship between the total PEDro score and time. The different colors of the bubbles represent the different PEDro scores: green represents a high PEDro score (greater than or equal tosix) and red represents a lower score (lower than six). A linear regression model was used to evaluate the development of methodological quality over time.

## Results

### Distribution of Scientific Literature

The included clinical trial dates ranged from 1982 to 2021. A total of *n* = 81 studies were included in the final analysis to evaluate the methodological quality over the past 40 years. Notably, no study was already listed in the PEDro database.

A total of *n* = 2 studies were retrieved between the years 1982 and 1989 (Squires and Buskirk, 1982; Wagner et al., 1986), *n* = 5 between 1990 and 1999 (Fulco et al., 1994; Koistinen et al., 1995; Naughton et al., 1995; Fulco et al., 1996; Taylor and Bronks, 1996), *n* = 18 between 2000 and 2009 (Casas et al., 2001; Takase et al., 2002; Bocqueraz et al., 2004; Shave et al., 2004; Choukèr et al., 2005; Friedmann et al., 2005; Heubert et al., 2005; Saito et al., 2005; Sandiford et al., 2005; Schiffer et al., 2005; Wehrlin and Hallen, 2006; Mackenzie et al., 2008; Richardson et al., 2008; Subudhi et al., 2008; Zhou et al., 2008; Richardson et al., 2009a; b; Wang and Chiu, 2009), *n* = 46 between 2010 and 2019 (Fukuda et al., 2010; Miyagawa et al., 2011; Basualto-Alarcón et al., 2012; Degache et al., 2012; Kroepfl et al., 2012; Maire r et al., 2012; Schommer et al., 2012; Faiss et al., 2013; Fan et al., 2013; Mairer et al., 2013; Sandfeld et al., 2013; Feriche et al., 2014; Ho et al., 2014; Julia-Sanchez et al., 2014; Kröpfl et al., 2014; Slivka et al., 2014; Trapp et al., 2014; DiPasquale et al., 2015; Seo et al., 2015; Brocherie et al., 2016; Girard et al., 2016; Shrestha and Singh, 2016; Filopoulos et al., 2017; Girard et al., 2017; Klenze et al., 2017; Lira et al., 2017; Lühker et al., 2017; Machado et al., 2017; Matu et al., 2017; Sweeting et al., 2017; Tymko et al., 2017; Wadley et al., 2017; Willis et al., 2017; Alhammoud et al., 2018; Cooke et al., 2018; Lee and Thake, 2018; Angeli et al., 2019; Charkoudian et al., 2019; da Mota et al., 2019; Gronwald et al., 2019; Lei et al., 2019; Morawetz et al., 2019a; Morawetz et al., 2019b; Mulliri et al., 2019; Sharma et al., 2019; Valenzuela et al., 2019), and *n* = 10 between 2020 and 2021 (Faulhaber et al., 2020; Jung et al., 2020; Limmer et al., 2020; Nell et al., 2020; Willis et al., 2020; De Groote et al., 2021; Kong et al., 2021; Magnani et al., 2021; Vasquez-Bonilla et al., 2021; Yamaguchi et al., 2021).

The mean PEDro score of the included studies over time was 5.1 ± 0.9. In the period from 1982 until 1989, only two studies were published in peer-reviewed journals, with a mean PEDro score of 4.5 ± 0.7. In the period from 1990 to 1999, five studies achieved a mean PEDro score of 5.0 ± 0.8. From 2000 to 2009, a total of n = 18 studies achieved a mean PEDro score of 4.6 ± 0.7, from 2010 to 2014 a total of *n* = 17 studies reached a score of 4.8 ± 0.9, and from 2015 to 2020 a mean PEDro score of 5.4 ± 1.0 was reached by *n* = 35 included studies. Four studies reached a mean PEDro score of 5.7 ± 0.5 out of 10 in the year 2021.

### Methodological Quality

**FIGURE 2:**
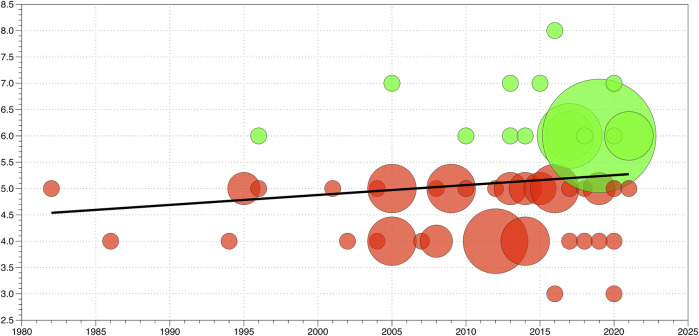
Bubble plot of the *n* = 81 included studies. Green dots represent studies with “good” methodological quality and a PEDro score ≥6 points. Red bubbles represent studies with lower PEDro scores of <6 points, indicating “fair” (4–5 points) or “poor” (<4 points) methodological quality. The size of the bubbles is related to the proportion of studies having the same PEDro score at a specific time point. The black line represents the linear regression line as a function of time.

The bubble plot in Figure 2 depicts the evolution of the PEDro scores of the included manuscripts from 1982 to 2021. Green bubbles represent a PEDro score of 6 and higher and red bubbles represent studies with a PEDro score of 5 or lower. The linear regression analyses demonstrated that 2.6% of the variance of the y-variable can be explained, and the linear regression line was calculated using the following equation: y = (0.0188 x year) + (−32.794). The slope of the linear regression line suggests that the mean PEDro score increases by 0.1 points each decade.

The methodological quality, measured with the PEDro score, ranged from 3/10 to 8/10, between the years 1982 and 2021. None of the included studies reached the maximum PEDro score of 10.

The highest achieved PEDro score in the assessed studies was a score of eight, which was awarded to an article published by one research group in the year 2016 (Brocherie et al., 2016). The second highest PEDro score was 7/10, which was achieved by four studies (Wehrlin and Hallen, 2006; Faiss et al., 2013; DiPasquale et al., 2015; Nell et al., 2020). A total of 23 studies achieved a PEDro score of 6/10. Total PEDro scores of six to eight, which are considered reflective of “good” methodological quality, were achieved in 34.6% (*n* = 28 studies) of all included studies (Cashin and McAuley, 2020).

**FIGURE 3:**
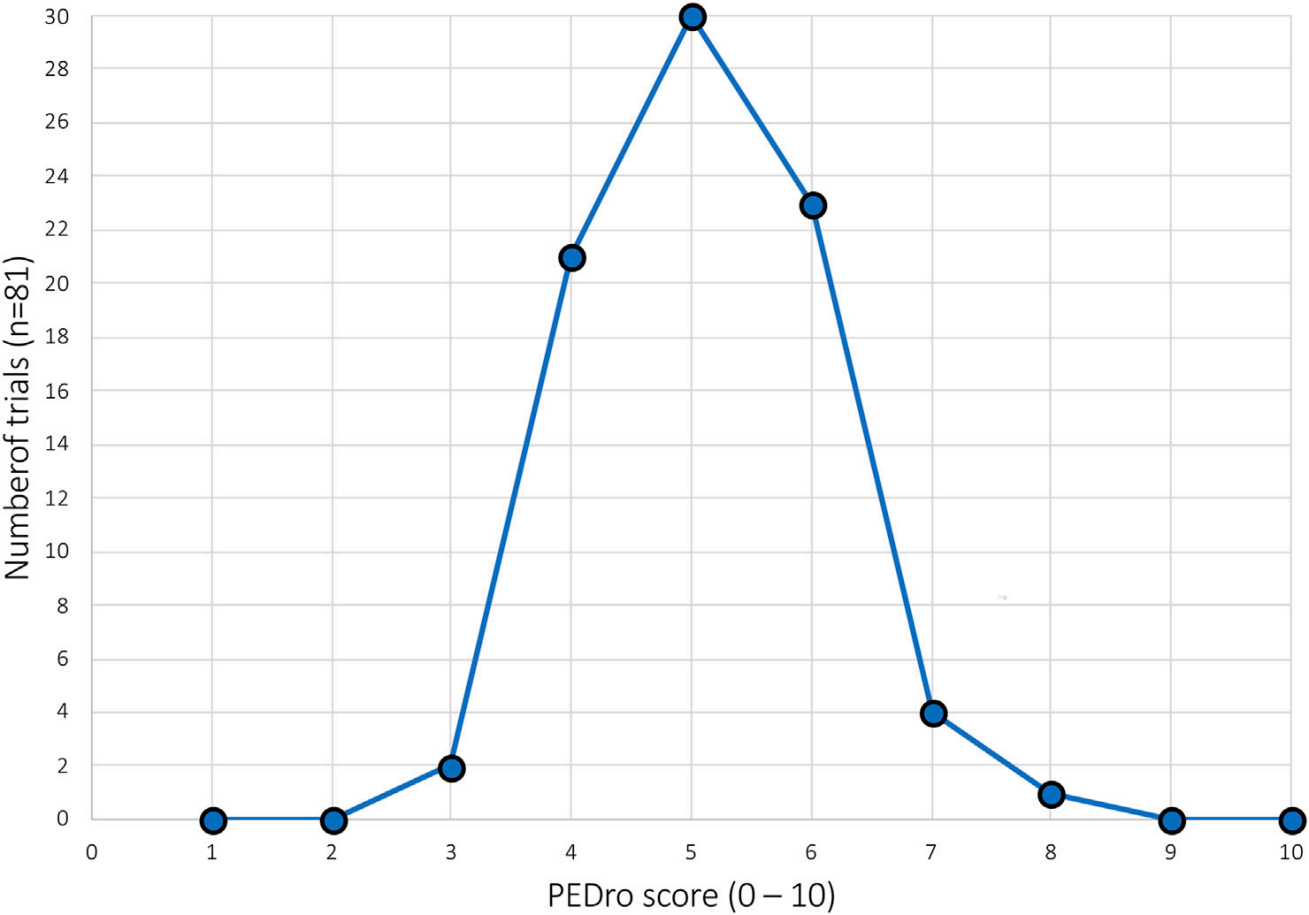
Absolute frequency distribution of the total PEDro scores of all included studies.

“Fair” methodological quality (PEDro score of four to five was achieved by 51 studies, reflecting 63.0% of the 81 included studies. Only 2.4% (2/81) of works are rated to have “poor” methodologic quality, which is considered a PEDro score of <4. A detailed overview of the number of studies and their PEDro scores is shown in Figure 3.

**FIGURE 4:**
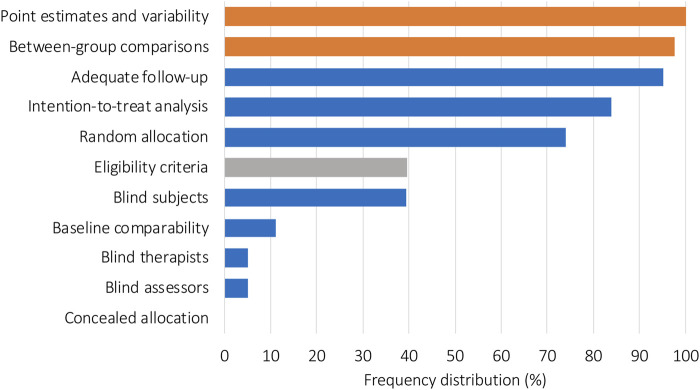
Relative frequency distribution of all included studies for each PEDro item. Orange bars represent items 10 and 11 of the PEDro score, which represent the interpretability of the data. Blue bars represent items 2–9, which are used to evaluate internal validity. The gray bar represents item 1, which evaluated external validity and was not used to calculate the total PEDro score.

Analysis of PEDro score single items can be seen in Figure 4. All included trials (100%) fulfilled the item for point estimates and variability, 97.5% included “between-group comparisons”, and 95.0% performed “adequate follow-up.” Lower relative frequency scores were observed for the items regarding “intention-to-treat analysis” (83.9%) and “random allocation” (74.0%). The items “eligibility criteria” (excluded from the total PEDro score ratings) and “blind subjects” each reached 39.5%. “Baseline comparability” (11.1%), “blind therapists and blind assessors” (each 4.9%), and “concealed allocation” (0%) had the lowest relative frequency scores.

## Discussion

This systematic review aimed to assess the methodological quality of clinical trials examining the physiological response to exercise under hypoxic conditions by considering PEDro scores. We also aimed to assess changes in the mean methodological quality over time.

The calculated mean PEDro score of the *n* = 81 included studies was 5.1 ± 0.9. Research groups that were able to blind the subjects and assessors (Brocherie et al., 2016) or subjects and therapists (Nell et al., 2020) had higher PEDro scores. Single- or double-blinding procedures are important to avoid bias. It has been demonstrated that trials without double-blinding yielded larger estimates of treatment effects than trials using double-blinding procedures (Schulz et al., 1995). From a theoretical perspective, studies that compared the effects of different partial pressures of inspired oxygen and barometric pressure could reach a maximum score on the PEDro scale (10/10). In particular, studies using closed chamber systems, where barometric pressure and the fraction of inspired oxygen can be controlled, are particularly predisposed to reaching the maximum methodological score on the PEDro score. It is nearly impossible to blind participants, therapists, or assessors, in case a study evaluates the difference between terrestrial altitude and a laboratory condition at the sea level. The use of mask systems or oxygen tents might help contribute to the blinding procedure, but barometric pressure can only be simulated in a closed chamber or at a real altitude. Studies that investigated the difference between terrestrial altitude (or did not have an environmental chamber system) and a laboratory setting at the sea level could only have reached a maximum PEDro score of 7/10 since the blinding of participants, therapists, and assessors could be a problematic issue in those setups. In the articles analyzed in the present study, only five works, representing around 6% of the included 81 studies, reached a PEDro score of seven or higher (Wehrlin and Hallen, 2006; Faiss et al., 2013; DiPasquale et al., 2015; Brocherie et al., 2016; Nell et al., 2020). Following the PEDro database, only these 6% could be considered to have good methodologic quality (PEDro, 1999; Cashin and McAuley, 2020). The modal PEDro score was 5 and the mean score was 5.1, suggesting that the overall methodological quality of these studies can be rated as “fair” (Cashin and McAuley, 2020).

Interestingly, none of the included studies concealed their sample allocation or failed to describe it based on their PEDro score. Random allocation is known to be an important method to ensure that the groups being compared are on an equivalent basis at the study start (Schulz, 2001). This point could be easily achieved because a point is awarded for this category even if it is not stated in the work that allocation was concealed. In this case, the study must state that allocation was via sealed opaque envelopes or that allocation involved contacting the holder of the allocation schedule who was “off-site” (PEDro, 1999). Only 11% of the included studies received a point for comparing groups that were similar at baseline for the most important prognostic indicators. Here, the ratings could be theoretically higher as at a minimum the report must describe at least one measure of the severity of the condition being treated and at least one (different) key outcome measure at baseline. The rater must be satisfied that the groups’ outcomes would not be expected to differ based on baseline differences in prognostic variables alone by a clinically significant amount (PEDro, 1999). However, on the other site, the meaningfulness of statistical testing for baseline differences has been questioned (Harvey, 2018).

Random allocation was satisfactory in 74% of the included studies. Although this number is relatively high, it should be considered with caution from a methodologic quality perspective. An article receives a point for this item just by mentioning that the allocation was random without further explanation. Good methods of generating a random allocation sequence include using a random-number table or a computer software program (Dettori, 2010). Less recommended methods to achieve random allocation are tossing a coin, drawing lots, or throwing a dice (Dettori, 2010). Quasi-randomization allocation procedures such as allocation by hospital record number, birth date, or alternation do not satisfy this criterion (PEDro, 1999).

The PEDro scale is used to assess the methodological quality of trials and to specifically identify those trials with good internal validity (PEDro items 2–9) that report enough data to make their results interpretable (PEDro items 10–11) (Moseley et al., 2020). Looking into the results section we assume that the results of the included studies can be rated as interpretable, as item 10 (between-group statistical comparison) and item 11 (point estimates and variability) achieved nearly perfect fulfillments. However, the specific items that define good internal validity demonstrate a wide range of scores among the included studies. Using the linear regression formula, it can be expected that “good” mean methodological quality as defined by a PEDro score of 6 (Cashin and McAuley, 2020) will be reached in the year 2063. However, this process could be accelerated, if upcoming studies in this area, especially consider items two to nine of the PEDro score. The number of published articles in the field of exercise under hypoxic conditions is increasing.

Our article not only demonstrates that the mean methodologic quality of studies is “fair” and increasing over time but also demonstrates that clear guidelines are needed to further increase the methodologic quality in the field. To increase internal validity, researchers should ensure careful study planning and implementation strategies. The results of our analysis demonstrate that adequate blinding procedures should be incorporated into studies whenever possible. However, as already mentioned, blinding procedures are now always easy to implement in studies investigating the effects of hypoxia, especially when no closed chamber systems are available or in the case the study is performed during terrestrial altitude. The results of the current analysis further show that future studies in this area are advised to realize concealed allocation and ensure baseline comparability, to increase their internal validity. It is important to mention that internal validity, the extent to which the design and conduct of a trial eliminate the possibility of bias, is a prerequisite for external validity (Moher et al., 2010). The effective use of external validity has the potential to speed up the implementation of worthwhile innovations and avoid unworthwhile efforts (Dyrvig et al., 2014).

It should be acknowledged that the PEDro score is only one tool for evaluating the methodological quality of clinical trials that is typically used (but not limited to) in current practice methods in physiotherapy. The use of the summary score from the PEDro scale has also been critically questioned, as it showed poor construct validity in addition to other limitations (Albanese et al., 2020). Other methodological quality assessment tools are available in the literature, such as the Cochrane risk of bias 2.0 tool, the EPOC risk of bias tool, and the CASP checklist, which can be found elsewhere (Ma et al., 2020). Researchers should therefore carefully choose and report the methodological quality assessment tool that they chose to use and try to achieve the highest score for their internal validity. A limitation of the current study might be that only studies written in English were used, and the inclusion of studies was limited to the aforementioned search strategies. Therefore, the current findings might over- or underestimate the true methodological quality of the entire current literature in this field.

## Conclusion

The mean PEDro score of trials investigating the difference between different hypoxic and normoxic conditions during exercise over the last 40 years is 5.1 ± 0.9, indicating “fair” methodological quality. This work’s linear regression showed a small positive trend toward higher scores in the future, with an increase of 0.1 point each decade. “Good” mean methodological quality in this research field can be expected in the year 2063 at the earliest given current trends. Although the results of the studies are interpretable, future studies in this field should incorporate adequate blinding procedures (if possible), concealed allocation, and baseline comparability. Future studies should consider including the relevant criteria during the planning of the study to achieve the highest possible methodological quality score.

## Data Availability

The original contributions presented in the study are included in the article/Supplementary Material; further inquiries can be directed to the corresponding author.
